# The Effect of the 5-HT_4_ Agonist, Prucalopride, on a Functional Magnetic Resonance Imaging Faces Task in the Healthy Human Brain

**DOI:** 10.3389/fpsyt.2022.859123

**Published:** 2022-04-12

**Authors:** Angharad N. de Cates, Marieke A. G. Martens, Lucy C. Wright, Cassandra D. Gould van Praag, Liliana P. Capitão, Daisy Gibson, Philip J. Cowen, Catherine J. Harmer, Susannah E. Murphy

**Affiliations:** ^1^Department of Psychiatry, University of Oxford, Warneford Hospital, Oxford, United Kingdom; ^2^Oxford Health National Health Service (NHS) Foundation Trust, Warneford Hospital, Oxford, United Kingdom; ^3^Oxford Centre for Human Brain Activity, Wellcome Centre for Integrative Neuroimaging, Department of Psychiatry, University of Oxford, Oxford, United Kingdom

**Keywords:** serotonin receptor 4, functional neuroimaging (fMRI), emotional processing, cognition, antidepressant

## Abstract

Depression is a common and often recurrent illness with significant negative impact on a global scale. Current antidepressants are ineffective for up to one third of people with depression, many of whom experience persistent symptomatology. 5-HT_4_ receptor agonists show promise in both animal models of depression and cognitive deficit. We therefore studied the effect of the 5-HT_4_ partial agonist prucalopride (1 mg daily for 6 days) on the neural processing of emotional faces in 43 healthy participants using a randomised placebo-controlled design. Participants receiving prucalopride were more accurate at identifying the gender of emotional faces. In whole brain analyses, prucalopride was also associated with reduced activation in a network of regions corresponding to the default mode network. However, there was no evidence that prucalopride treatment produced a positive bias in the neural processing of emotional faces. Our study provides further support for a pro-cognitive effect of 5-HT_4_ receptor agonism in humans. While our current behavioural and neural investigations do not suggest an antidepressant-like profile of prucalopride in humans, it will be important to study a wider dose range in future studies.

## Introduction

Globally, more than 300 million people are affected by depression at any one time (1), and up to one in five people experience at least one lifetime episode (2). Approximately two thirds of patients respond to first-line antidepressant treatments (3), although it is typical to experience a delay before therapeutic benefit (4). However, up to one third of patients do not achieve remission with antidepressants, but rather develop a treatment-resistant illness, with significant impact on their occupational and social functioning (2). Within this context, there is a pressing need to develop new treatments for depression that target novel mechanisms and have a faster onset of therapeutic action.

Prucalopride acts selectively on 5-HT_4_ receptors as a partial agonist. These post-synaptic receptors are located in regions of the brain linked with both cognition and emotional processing, including the limbic system (including the anterior cingulate cortex), basal ganglia (including the putamen), and neocortex (5–7). Evidence from animal studies suggests that 5-HT_4_ receptor agonism may be a promising new target for rapid acting antidepressant action (4). In contrast to the delayed action of conventional antidepressants, 5-HT_4_ receptor agonists acutely increase the firing rate of midbrain serotonergic cells, *via* their action on medial prefrontal cortical pyramidal cells (8–10). In addition, 5-HT_4_ receptor agonism rapidly induces the production of neurotrophic proteins required for neuroplasticity, such as brain derived neurotrophic factor, an outcome typically only seen with chronic antidepressant treatment (11).

Consistent with this, animal behavioural studies have demonstrated that 5-HT_4_ receptor agonists are rapidly active in animal models of depression, ameliorating the anhedonia-like behaviours produced by chronic mild stress and corticosteroids (11–13). In addition, 5-HT_4_ receptor agonists have been shown to have pro-cognitive effects, and in particular facilitate hippocampal dependent learning and memory processes (14–16), which may indicate clinical uses across a variety of mental illness diagnoses, including anxiety and schizophrenia. Consistent with this, 5-HT_4_ receptor agonists have been shown to lead to rapid anxiolysis in rodents (8, 11, 17), which may relate to direct activation of medial prefrontal cortex 5-HT_4_ receptors (8). They also have been suggested as potential neuroprotective agents in schizophrenia as subfield volumetric reduction in the hippocampus in patients with first episode psychosis appears to correlate with 5-HT_4_ receptor density (18).

Investigating the neuropsychological effects of 5-HT_4_ receptor agonism in humans has been challenging due to the side-effect profile of early agents. However, the 5-HT_4_ agonist prucalopride, which is licenced for the treatment of constipation, has recently been successfully used as a probe of human 5-HT_4_ function (19, 20). Prucalopride is a selective high-affinity 5-HT_4_ partial agonist with good brain penetration (21) and a pro-cognitive and antidepressant-like profile in animal models (12, 22). We have previously shown that prucalopride (1 mg, for one and 6 days) has a pro-cognitive effect on memory tasks in healthy volunteers (19, 20), and increases hippocampal and angular gyrus function during memory processing (20). However, it is currently not known whether prucalopride also has an antidepressant-like profile in humans.

Conventional antidepressants work rapidly both behaviourally and neurally to decrease processing of negative information, and increase responses to positive stimuli (23–27). Selective serotonin reuptake inhibitors (SSRIs) have been shown to decrease amygdala responses to fearful faces in healthy participants (28, 29), and also in people with depression (27). Experimental medicine studies based on this model can provide a rapid and reliable detection of drugs with clinical antidepressant activity (30–32), and therefore provides a useful translational approach to screen novel compounds in humans for potential antidepressant activity.

Here, we examine whether short-term (6–7 days) administration of prucalopride, affects neural and behavioural emotional processing in healthy human volunteers. We hypothesised that prucalopride would decrease the neural response to negative emotional information similar to that previously observed with serotonergic antidepressants (i.e.) decreased response to negative vs. positive emotional stimuli in a network including the medial prefrontal cortex (including the anterior cingulate cortex), orbitofrontal cortex, amygdala, and hippocampus in an fMRI paradigm.

## Materials and Methods

### Participants

**FIGURE 1:**
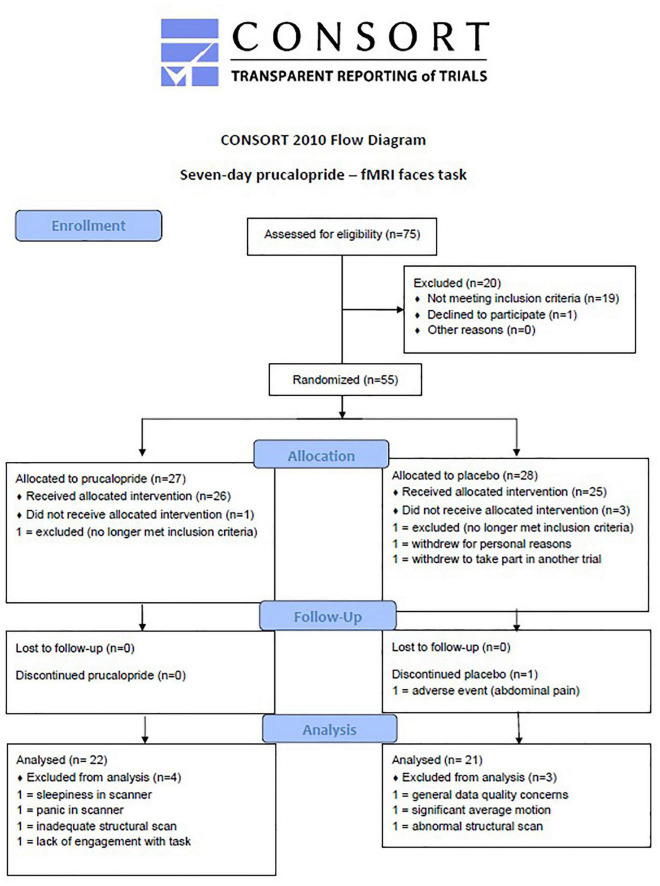
CONSORT diagram to show flow of participants through the study. CONSORT diagram to show flow of participants through the study.

Right-handed healthy participants (*N* = 50, aged 18–40) were recruited and randomised to either prucalopride [7 days × 1 mg (imaging taking place on day 6)] or placebo, in a double-blind randomised design. Participants were all fluent in English and had no contraindications to prucalopride. Participants were young adults of any gender, healthy, right-handed, not pregnant or breast feeding, and with a BMI between 18 and 30 (full inclusion and exclusion criteria are included in Supplementary Material). We excluded any potential participant who may be more at risk of side effects with prucalopride (e.g., chronic bowel disease). The study was approved by the University of Oxford Central University Research Ethics Committee (MSD-IDREC reference R57219/RE001) and the protocol was pre-registered with clinicaltrials.gov (NCT03572790). This is a separate part of the study as reported in Ref. (20) and involves the same participants. No changes to methods occurred after start of the study and the flow of participants is outlined in Figure 1. Participants gave written informed consent.

### Design and Randomisation

The study had a between-subject, double-blind, placebo-controlled design. Participants were assigned in a random manner to 7 days of prucalopride (Resolor) 1 mg daily or placebo (lactose tablets, Rayonex Biomedical) in a 1:1 allocation. Randomisation was blocked in design (block size = 4), stratified for sex and undertaken with an online tool (sealedenvelope.com on June 1, 2018). Group allocation was concealed from participants, investigators and assessors using sequential numbered containers, and the encapsulation process ensured that both capsules appeared identical. All capsules were consumed by participants at home.

Participants underwent a 3T scan including fMRI tasks on day 6 (Research Visit 1): a structural T1 scan, a faces emotion recognition test task, a memory encoding task, an arterial spin labelling (ASL) scan, and a resting state scan (further details of the scanning protocol can be found here: 10.5281/zenodo.6107724). By day 6 when imaging was carried out, prucalopride would be expected to be at a steady state (terminal half-life approximately 24 h) (33). Testing occurred at the Department of Psychiatry and the Oxford Centre for Human Brain Activity (OHBA), part of the Wellcome Integrative Neuroimaging Centre (WIN). For female participants testing/scanning did not occur during the premenstrual week.

### Questionnaire Measures

To obtain baseline measures of mood, anxiety, and personality, self-report questionnaires were completed: Beck Depression Inventory-II (34); Snaith-Hamilton Pleasure Scale (SHAPS) (35); Spielberger State-Trait Anxiety Inventory, Trait Version (STAI-T) (36); and Eysenck Personality Questionnaire (37). Affect and anxiety were measured three times [baseline (screening), pre-imaging (day 6), and post-imaging (day 6)] using: the Positive and Negative Affect Scale (PANAS) (38); visual analogue scales (VAS) (39); and the Spielberger State-Trait Anxiety Inventory, State Version (STAI-S) (36). Commonly reported side effects were also measured at these three time-points using a scale where participants rated the extent to which they were experiencing any of these (40). At the end of the study, participants were asked to guess their group allocation with a forced-choice question.

### Functional Magnetic Resonance Imaging Faces Task

In the fMRI emotional processing task (41) (see Supplementary Figure 1), participants were told that the aim of the task was to correctly identify the gender of each face shown in the images (male or female), with no reference made to the face emotion. This task was modified from previous versions used within our laboratory (27, 32) with block lengths and orders optimised for fMRI [see Ref. (42), full details are in Supplementary Material]. Briefly, faces were presented rapidly for 100 ms in isolation and participants had to respond to indicate the assumed gender of the face as quickly as possible using a button press. The task followed an A-B-Rest design, where a block of condition A (fearful faces, 18 s) was followed by a block of condition B (happy faces, 18 s) then a block of rest (12 s). There was an additional rest block at the start of the experimental run. There were seven repeats of each block, generating a total 126 s of each emotion condition and 96 s of rest. Each block consisted of six trials: three male and three female images presented in a randomised order. Each image was shown once over the length of the experiment. Previous versions of this task have been shown to be sensitive to the acute effects of antidepressants on neural processing (32). Face images for the task were modified from the Karolinska Directed Emotional Faces (KDEF) set.^1^ The task software was written using Psychopy version 1.84.2.

### Demographic and Behavioural Data Analysis

Analysis of these data occurred as previously described in Ref. (20). Demographic characteristics and baseline clinical measures were analysed using independent sample *t*-tests (continuous variables), Fisher’s exact tests (native language and sex) and logistic regression (education). To assess changes in subjective mood and side effects before and after prucalopride/placebo administration, repeated measure analyses of variance (ANOVAs) were performed with time as a within-subject factor (baseline, pre-testing Research Visit 1). Repeated measures analyses of variance (ANOVAs) were used to analyse fMRI behavioural task data with a between-subjects factor of the treatment group (prucalopride or placebo) and within-subjects factor of gender accuracy with face emotion (fear or happy). *Post hoc* analyses using independent samples *t*-tests were performed to follow-up interactions observed. Levene’s test (*t*-tests) and the Greenhouse-Geisser procedure (ANOVAs) were used as appropriate, correcting degrees of freedom where equal variances between groups could not be assumed. A *p*-value less than 0.05 was used to denote statistical significance. Partial eta squared are reported as a measure of effect size. Behavioural data were analysed in SPSS (version 25, IBM). Graphs were produced using GraphPad Prism (version 9.3.1) and Excel (version 2016). Management of outliers on behavioural tasks is discussed in Supplementary Material.

### Magnetic Resonance Imaging Data Acquisition and Analysis

Blood-oxygenation-level-dependent (BOLD) fMRI and T1-weighted anatomical images were acquired using a 3-Tesla Siemens Prisma scanner, equipped with a 32-channel head matrix coil (Siemens, Erlangen, Germany). Here we report results from the faces task. Participants also completed an encoding memory task and an arterial spin labelling (ASL) during the scan, the details of which have been published previously (20) with cerebral blood flow data also included here as a covariate. Results from the resting state scan will be reported elsewhere. Foam padding and a head restraint were used to control head movement. Further details of fMRI and structural Magnetic Resonance Imaging (MRI) acquisition can be found in the Supplementary Material. The full acquisition protocol, along with radiographic procedure is available from the Open WIN MR Protocols database here: 10.5281/zenodo.6107724.

Faces imaging data were analysed with FSL.^2^ fMRI data were pre-processed and analysed using FEAT (FMRI Expert Analysis Tool), version 6.0.4, part of FSL (FMRIB’s Software Library; see text footnote 2). For further information on the pre-processing, first-level and second-level analyses, and confirmatory analyses, please refer to the Supplementary Material.

Pre-processing involved various steps designed to reduce noise-related variability in the data and to improve the validity of the statistical analysis. Each participant’s imaging data underwent the following steps: (1) Removal of non-brain structures using BET, (2) motion correction using MCFLIRT, (3) spatial smoothing using a Gaussian kernel of FWHM 5 mm, (4) grand-mean intensity normalisation of the entire 4D dataset by a single multiplicative factor and high-pass temporal filtering cut-off = 90 s (Gaussian-weighted least-squares straight-line fitting, with sigma = 45 s), and (5) B0 unwarping using fieldmap rads and magnitude images for distortion correction.

In the first-level analysis, individual activation maps were computed using the general linear model with local autocorrelation correction. Three explanatory variables were modelled: “happy” and “fear” images and explicit fixation cross. Temporal derivatives were included in the model. Variables were modelled by convolving each block with a haemodynamic response function, using a variant of a gamma function (i.e., a normalisation of the probability density function of the gamma function) with a standard deviation of 3 s and a mean lag of 6 s. No included participant demonstrated significant movement: absolute displacements were less than 1 voxel and relative displacements less than 1/2 voxel. At the whole-brain level, fearful images were contrasted with happy and fixation cross, resulting in the following model: (1) fear > fixation; (2) fear < fixation; (3) happy > fixation; (4) happy < fixation; (5) fear > happy; (6) happy < fear; (7) mean (of happy and fearful faces) > fixation; (8) mean < fixation.

In the second-level analysis, whole-brain individual data were combined at a group level (placebo vs. prucalopride) using a mixed-effects group cluster analysis across the whole brain corrected for multiple comparisons, and cerebral blood flow and grey matter maps as covariates of no interest. The potential effect of sex was considered as a covariate of interest in sensitivity analyses. Groups were contrasted with each other using the following comparisons: (1) placebo > prucalopride; (2) prucalopride > placebo; (3) placebo mean; (4) prucalopride mean; (5) mean of all participants. Brain activations showing significant group differences were identified using cluster-based thresholding (*Z* > 3.1, *p* < 0.05 corrected). Significant interactions from whole-brain analyses were further explored by extracting percentage BOLD signal change for each type of contrast. As the amygdala and prefrontal cortex were a particular focus of our hypothesis, the amygdala, anterior cingulate cortex (ACC), medial frontal cortex (MFC), and orbitofrontal cortex (OFC) were pre-specified as regions of interest (ROI). A structural small volume correction analysis was run using the Harvard-Oxford subcortical atlas at a 90% threshold. A functional ROI mask was also created for the left and right of each ROI for the mean effect of task by multiplying mean activation for all participants by the Harvard-Oxford subcortical atlas anatomical mask at a 50% threshold, and percentage BOLD signal change for each contrast in each hemisphere was extracted in order to identify the profile of drug effect. All activations are reported using MNI co-ordinates.

FSLVBM and Oxford ASL was conducted as detailed in Ref. (20). ACC and amygdala perfusion between groups was compared using fslmeants: parameter estimates of perfusion were extracted from resting perfusion maps (previously computed using Oxford_ASL in units of ml/100 g/min) using anatomical Harvard–Oxford masks of the ACC and the left and right amygdala (amygdala at a 50% threshold).

## Results

### Participants

50 (100% of target) participants were recruited between June 11, 2018 and May 17, 2019. One participant was excluded from all analyses for data quality concerns raised at the time of data collection. Two other participants were excluded from fMRI analyses for persistent sleepiness and acute anxiety during the scan. A further four participants were excluded from fMRI analyses for (i) a structural brain variant that affected registration to standard space, (ii) a poor quality structural scan according to MRIQC assessment, (iii) significant motion during the scan, and (iv) lack of engagement with the faces task determined from behavioural responses.

Analysis occurred in originally assigned groups. The final groups for behavioural analysis (*N* = 43, 21:22 = placebo: prucalopride, aged 18–36) were well matched for age, BMI, level of education, use of substances, and NART scores (see Supplementary Table 1). However, compared to those with English as a first language, for non-native English speakers the odds of being in the prucalopride group were 0.11 (95% CI 0.02–0.59, *p* < 0.001). Randomisation guesses (data missing for one participant) suggested that participants were better than chance at guessing group allocation, particularly in the placebo group (correct guess: placebo 80.0%, prucalopride 69.6%; data missing for one participant in the placebo group). As previously reported (20), there were no adverse events in the prucalopride group; one person discontinued placebo due to abdominal discomfort.

### Questionnaire Results

As reported previously in the full sample (20), there were no significant differences in state anxiety or affect between the prucalopride and placebo group (all *p*s > 0.5, see Supplementary Table 2 for updated results specific to this sample). There were also no significant differences in reported side effects at baseline or during the study visits and only minimal side effects were present for headache, decreased appetite, flatulence, fatigue and gastrointestinal sounds (all *p*s > 0.3).

### Behavioural Results of Functional MRI Behavioural Faces Task

**FIGURE 2:**
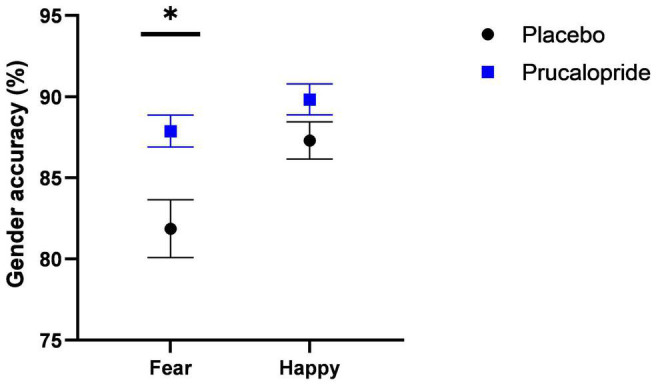
Behavioural results of fMRI faces task. Accuracy (%; unadjusted) to gender discrimination task as part of fMRI faces paradigm in placebo vs. prucalopride group. Error bars show standard error of the mean. Black circle = placebo; blue square = prucalopride. * = *p* < 0.05 (*T* test; *N* = 43; 21:22 = placebo: prucalopride).

There was a significant main effect of group [*F*(1,41) = 7.47, *p* = 0.009, np^2^ = 0.15], reflecting improved accuracy in the prucalopride group compared with the placebo group [accuracy (%): prucalopride *M* = 88.85, SEM = 0.80; placebo *M* = 84.58, SEM = 1.36]. There was also a significant emotion × group interaction [*F*(1,41) = 4.48, *p* = 0.04, np^2^ = 0.10]. This interaction was driven by significantly increased accuracy for fearful faces in the prucalopride group compared with the placebo group [*t*(1,31.41) = −2.955, *p* = 0.006] but no significant group differences in the accuracy for happy faces [*t*(1,41) = −1.702, *p* = 0.096], see Figure 2. These results were unchanged by adjusting for non-responses [main effect of group: *F*(1,41) = 8.91, *p* = 0.005, np^2^ = 0.18; emotion × group interaction: *F*(1,41) = 6.91, *p* = 0.012, np^2^ = 0.14]. There was no difference in average reaction speed between the groups (*p* = 0.39).

### Faces Task Functional MRI

#### Main Effect of Task

**FIGURE 3:**
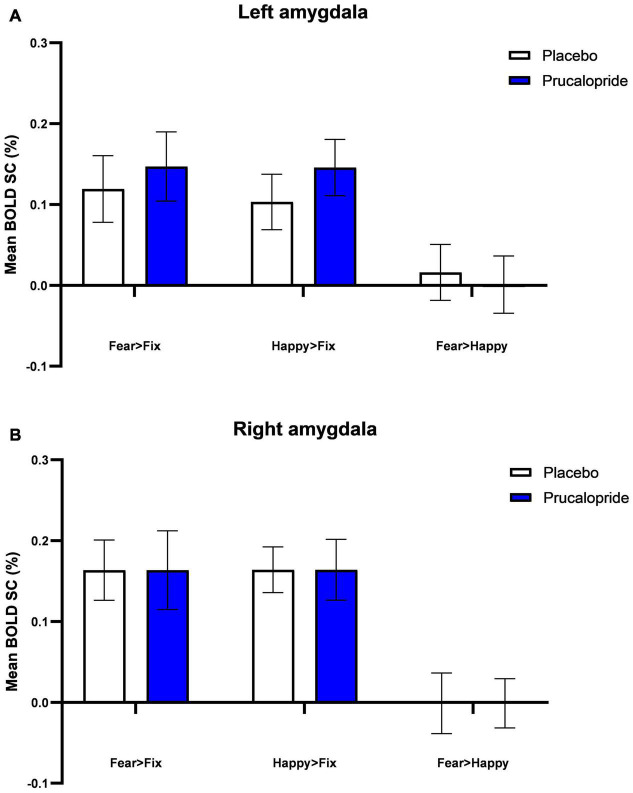
Blood-oxygenation-level-dependent percentage signal change (SC) extracted from left amygdala **(A)** and right amygdala **(B)**. Group mean of BOLD percentage signal change extracted from L amygdala **(A)** and R amygdala **(B)** in response to fearful and happy images (fear > fixation; happy > fixation; fear > happy). Error bars show standard error of the mean. The anatomical mask used was the Harvard-Oxford atlas at 90% threshold.

In order to determine if the functional MRI (fMRI) task engaged brain regions previously associated with fearful and happy facial stimuli, blood-oxygen-level-dependent (BOLD) activation in response to fearful faces and happy faces and the mean of both valences was compared to the baseline (i.e., fixation cross) across both groups (see Supplementary Figure 2). As per previous data, significant brain activations were observed in a network of regions (43–45), including the superior, middle and inferior frontal gyrus, paracingulate gyrus, frontal pole, pre-central gyrus, left and right putamen, left insular cortex, and left thalamus. These findings confirm that the task engaged brain areas implicated in the processing of both fear and happiness, similar to previous work with this version of the task (42). Unexpectedly, we did not see differential whole-brain activation related to face emotion (see Supplementary Figure 2), and there was a similar level of amygdala activation to both fearful and happy faces bilaterally (see Figure 3). For this reason, we present the mean effect of task [mean (of fear + happy) > fixation] as our primary analysis, with reference to results relating to emotional valence as appropriate.

#### Effect of Treatment–Whole Brain Analysis

**FIGURE 4:**
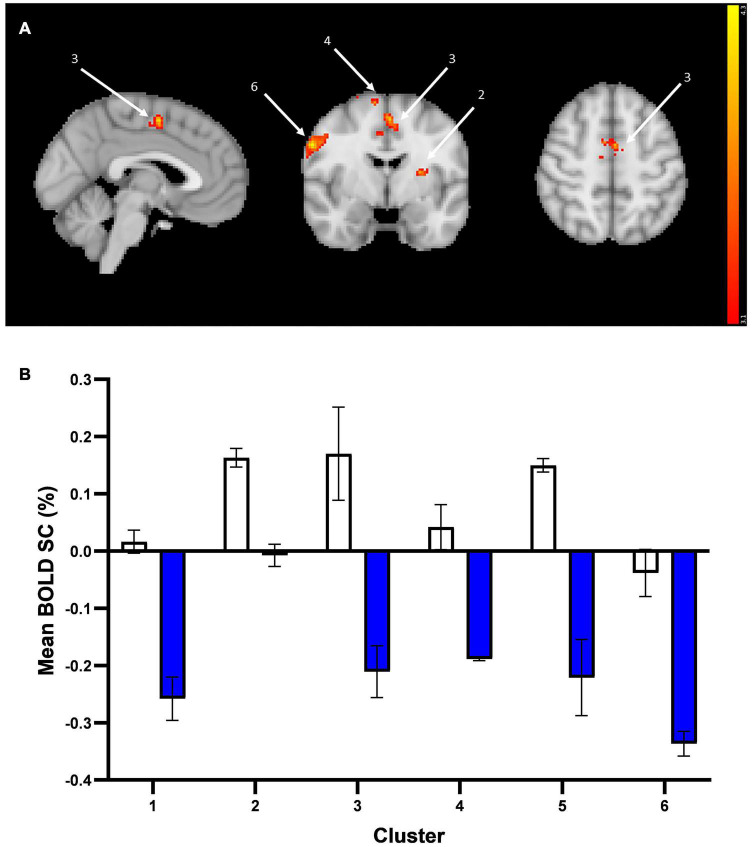
Whole brain fMRI Faces Task results. **(A)** Whole-brain activation in response to mean effect of task (mean > fixation) in placebo vs. prucalopride group. Sagittal, coronal, and axial images (shown at MNI location 45,61,62) depicting significantly increased activation in the placebo group for the mean contrast in significant clusters (Clusters: 1 = L post-central/pre-central gyrus; 2 = L putamen/insula; 3 = anterior cingulate cortex; 4 = R superior frontal gyrus; 5 = L supramarginal/post-central gyrus; 6 = R pre-central/post-central gyrus); cluster 1 and 5 not visible in figure. Images thresholded at *z* > 3.1 *p* < 0.05 corrected. Red to yellow colours identify increases in brain activation (scale *Z* = 3.1–4.3). **(B)** Group mean of BOLD percentage signal change (SC) extracted from the 6 clusters (mean > fixation). Error bars show standard error of the mean. White = placebo; blue = prucalopride. Clusters: 1 = L post-central/pre-central gyrus; 2 = L putamen/insula; 3 = anterior cingulate cortex; 4 = R superior frontal gyrus; 5 = L supramarginal/post-central gyrus; 6 = R pre-central/post-central gyrus.

When averaging the two emotions (vs. baseline), there was altered activation in the prucalopride group in 6 clusters (i) right pre-central/post-central gyrus, (ii) left supramarginal/post-central gyrus, (iii) right superior frontal gyrus, (iv) anterior cingulate cortex (ACC), (v) left putamen/insula, (vi) left pre-central/post-central gyrus (see Figure 4A and Table 1 for details of clusters). Figure 4B represents the group-level extracted BOLD signal change for these clusters. Across all clusters, the prucalopride group showed reduced activation in this network of areas.

**TABLE 1:** Details of activation clusters (placebo > prucalopride) from whole brain analysis for the mean effect of task.

Cluster number	Size (voxels)	*Z*-max	*p*-value	*Z*-max location (MNI)	Main regions involved in cluster
6	408	4.56	<0.001	62,−2,32	R pre-central/post-central gyrus
5	299	4.6	<0.001	−62,−30,38	L supramarginal/post-central gyrus
4	242	3.65	<0.001	12,2,68	R superior frontal gyrus
3	178	2.71	0.002	6,0,50	Anterior cingulate cortex
2	116	1.68	0.021	−28,−6,12	L putamen/insula
1	106	1.5	0.032	−48,−18,42	L post-central/pre-central gyrus

More specifically, as shown in Figure 4B, the left and right pre-central/post-central gyrus and right superior frontal gyrus clusters showed deactivation in the prucalopride group versus no or little activation in the placebo group; the left putamen/insula cluster showed activation in the placebo group versus no activation in the prucalopride group; the ACC and left supramarginal cluster showed activation in the placebo group versus deactivation in the prucalopride group.

#### Effect of Treatment–Region of Interest Analysis

There were no significant group differences in response to fear vs. happy facial expressions, or the mean effect of task, in the medial frontal cortex, orbitofrontal cortex, or left or right amygdala. Examining the effect of prucalopride versus placebo on the left and right amygdala to fearful faces demonstrated that prucalopride did not reduce activation in the amygdala as is typically seen with serotonergic antidepressants (28); instead there was no difference in amygdala activation between the placebo and prucalopride groups (see Figure 3).

**FIGURE 5:**
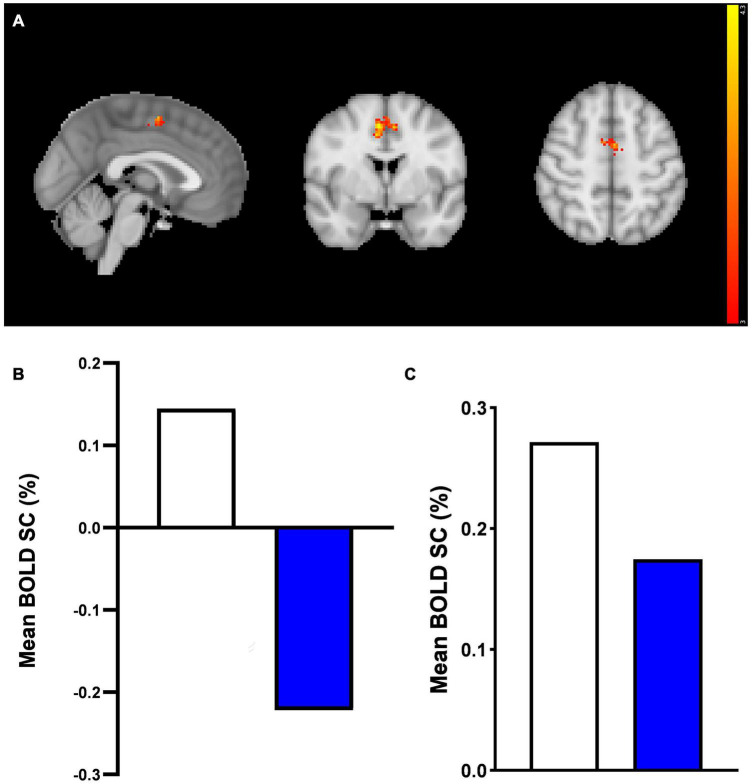
Anterior cingulate cortex region of interest fMRI faces task results. A region of interest activation for the anterior cingulate cortex (ACC) in response to mean effect of task in placebo vs. prucalopride group. Sagittal, coronal, and axial images (shown at MNI location 45,63,62) depicting significantly increased activation in the placebo group for the mean > fixation contrast [placebo > prucalopride, *Z* = 4.39, *p* < 0.0002, peak voxel location: *x* = 6, *y* = 0, *z* = 50, cluster size = 164 voxels]. Images thresholded at *z* > 3.1, *p* < 0.05 corrected. Red to yellow colours identify increases in brain activation (scale *Z* = 3.1–4.3). ACC Mask = Harvard Oxford atlas. **(B)** Group mean of BOLD percentage signal change (SC) extracted from the cluster in **(A)**. White = placebo, blue = prucalopride. **(C)** Group mean of BOLD percentage signal change (SC) extracted from functional anterior cingulate cortex (ACC) mask in response to mean effect of task. A functional ROI (SVC) mask was created for the ACC for mean > fixation by multiplying mean activation for all participants by the anatomical mask. White = placebo, blue = prucalopride.

However, in the ACC, consistent with whole-brain results, the prucalopride group showed deactivation compared with little activation in the placebo group in response to the mean effect of task (mean faces) [placebo > prucalopride, *Z* = 4.39, *p* < 0.0002, peak voxel location: *x* = 6, *y* = 0, *z* = 50, cluster size = 164 voxels] (see Figure 5A for cluster and Figure 5B for group-level extracted BOLD signal change). This pattern of reduced activation in the prucalopride group compared to the placebo group was unchanged when we performed a region of interest small volume correction analysis using a functional mask [using the same method as Ref. (20)] (Figure 5C).

### Analyses Controlling for Potential Confounds

As previously reported in Ref. (20), there were no group-related differences in grey matter between groups (FSLVBM: placebo > prucalopride, *p* = 0.91; prucalopride > placebo, *p* = 0.44). There was also no difference between groups in either global blood flow [Oxford_ASL: *p* = 0.64 (grey matter), *p* = 0.60 (white matter)] or regional blood flow (placebo > prucalopride *p* = 0.71; prucalopride > placebo *p* = 0.38). Whole brain and ROI results were similar with and without correction for cerebral perfusion and grey matter maps, and sex (see Supplementary Figure 3). Left and right amygdala and ACC perfusion did not differ between groups: L&R amygdala smoothed data *p* = 0.65 (L), *p* = 0.87 (R); ACC smoothed data *p* = 0.68 (see Table 2). Native language and overall accuracy to gender discrimination during the scan were not correlated using linear regression (*R*^2^ = 0.012).

**TABLE 2:** Left and right amygdala and anterior cingulate cortex (ACC) perfusion (ml/100 g/min).

		Left amygdala	Right amygdala	Anterior cingulate cortex
Smoothed (2.12)	Placebo	41.7	42.1	51.0
	Prucalopride	42.6	41.8	51.9
Unsmoothed	Placebo	41.7	42.0	52.0
	Prucalopride	42.7	41.8	52.9

## Discussion

The main finding of our study is that after 6 days of prucalopride treatment, healthy young adults were more accurate at identifying the gender of faces in a rapidly presented faces task compared to the placebo group, particularly in the context of fearful faces. In addition to this improved behavioural performance, we saw a reduction in brain activity in the prucalopride group, relative to the placebo group, in response to fearful and happy faces in a network of regions, including the medial prefrontal cortex (mPFC) [anterior cingulate cortex (ACC), superior frontal gyrus], and inferior parietal lobule (supramarginal gyrus). This reduction in activation in the ACC during the task was reproduced at both the whole-brain and region of interest level.

The improvement in accuracy of gender discrimination seen in the prucalopride group is consistent with our previous work demonstrating a pro-cognitive effect of acute and 6 day prucalopride administration in healthy volunteers (19, 20). Whilst previously we have reported that prucalopride acts to increase learning and memory in recall and recognition tasks, here we see this prucalopride-related improved performance extends to a simple perceptual discrimination task. Interestingly, this improved accuracy in the prucalopride group specifically related to fearful faces rather than happy, suggesting that prucalopride may have allowed participants not only to focus better on the task but also be less distracted by fearful faces compared to participants in the placebo group.

The rapid, specific, and potentially chronic improvements seen in learning and memory with 5-HT_4_ receptor agonists in rodents (12, 14, 46, 47) have been suggested to occur secondary to an increase in neurotransmitters and neurotrophic proteins, perhaps particularly acetylcholine release (48, 49), which result in downstream effects including neuroplasticity (17, 46). A recent study has also shown that prucalopride reduces bursts of AMPA receptor-mediated currents in the hippocampus, altering glutamatergic transmission (50) and making it a particularly interesting candidate for enhancing cognition. However, there is less understanding from the animal literature in terms of the impact of 5-HT_4_ receptor agonism on cognitive processes involved in the discrimination task used here, which could include effects on attention, amongst others.

This improved behavioural performance was accompanied by reduced activation in a network of regions, including the mPFC and inferior parietal lobule. Interestingly, this network overlaps with regions that form the default mode network (DMN). The default mode network (DMN), also known as the medial frontoparietal network (M-FPN) is a connected network of brain regions that experience greater activity at rest than during performance of externally orientated active cognitive tasks (51). Abnormalities of the DMN are present in cognitive impairment–both in Alzheimer’s (52) and those at high risk of dementia [i.e., mild cognitive impairment (MCI) (53)]–but also in depression, where abnormalities may pose a cognitive risk factor for recurrent illness (54). Abnormal activity within the DMN is classically linked to increased rumination, but is also associated with impaired control of attention (54). Failing to reduce activation within the DMN whilst actively undertaking a cognitive task appears to directly relate to poor task performance, whereas appropriate “switching off” of the DMN during tasks is linked with improved cognitive performance (55). In this way, improving appropriate deactivation of the DMN during cognitive tasks has been proposed as a transdiagnostic marker to assess for pro-cognitive effects of interventions (55). Therefore, the decreased activation of DMN-related regions during task performance in this fMRI faces task may reflect enhanced attention and cognitive control, which is consistent with the improvement in performance accuracy seen behaviourally.

Contrary to our hypothesis, we did not see a clear antidepressant-like profile of prucalopride on the emotional processing task. In humans, in both healthy participants and depressed patients, antidepressants work rapidly to decrease brain regional activity involved in processing of negative information, and increase activity arising from positive stimuli (23–26). These effects occur prior to any significant mood change in patients with depression. This emotional processing model has been shown to provide a rapid and reliable detection of drugs with clinical antidepressant activity (30, 31), and thus provides a feasible methods to screen 5-HT_4_ agonists in humans for potential antidepressant activity. Results from this study are not consistent with prucalopride demonstrating an antidepressant effect. That is, we saw a general decrease in activity in response to faces rather than an emotion specific effect (i.e.) there was no reduction in amygdala activation to fearful faces with prucalopride as seen with SSRIs (28, 56). This is a similar finding to an earlier behavioural study in healthy participants in our laboratory where we found no evidence of an antidepressant profile of prucalopride on behavioural tasks of emotional processing following a single 1 mg dose (19).

Given the consistency of evidence from animal studies demonstrating an antidepressant-like profile of 5-HT_4_ receptor agonism on a number of models of depression, it is important to consider why this effect has not yet translated to human models. Although animal models are vital in drug discovery, they are frequently poorly predictive of clinical efficacy in humans (57). However, we also need to consider possible other explanations for the lack of antidepressant-like profile seen in the current study. Importantly, the version of the fMRI faces task used in this study did not elicit differential activity in the amygdala in response to “happy” and “fearful” faces in this sample, unlike previous versions of the task. We believe that this may have related to enhanced standardisation of the face images between emotion conditions, which was intended to minimise the confounding differences such as hair position, face size and image luminance, and provide a more constrained contrast between the emotions. In this sample it may have led to a perception that happy faces were more threatening, and it also appears to have made the task more cognitive challenging (participants had generally reduced accuracy than seen with previous versions of this task; see Supplementary Material). Within this context it is necessary to be cautious in interpreting the apparent lack of an antidepressant effect, a finding which needs to be replicated using a task that is more successful in probing emotion-specific responses to face stimuli. Furthermore, a low dose of prucalopride (1 mg is half the licenced dose of 2 mg) was used in the current study to minimise gastrointestinal side effects. It is possible that this dose is insufficient to produce positive effects on emotional processing, and we currently lack information about the appropriate dose of prucalopride needed for optimal occupancy of brain 5-HT_4_ receptors. We calculated the sample size needed (17 per group to give 90% power with α = 5%) on the basis of a conservative estimated effect size of 0.5–0.7, but we aimed for oversampling of 25 per group to account for fMRI analyses. Unfortunately, three participants were excluded prior to fMRI analysis for technical reasons, and a further four were excluded during analysis for fMRI-related concerns or a lack of engagement with the task (determined from behavioural responses), which likely reduced the statistical power of the study. Due to chance, most participants whose first language was not English received placebo, but the task was not verbal in nature, and there was no evidence that first language and task performance were correlated. We also note that our findings relate to prucalopride specifically, and there may be subtle variations in the effects of other 5-HT_4_ receptor agonists (58). However, there is no evidence that there are marked variations between different 5-HT_4_ receptor agonists in the brain regions of interest in this study.

In conclusion, the behavioural results from this fMRI faces task provide further evidence that prucalopride can enhance cognition in healthy participants. This is consistent with, and extends, our previous findings of pro-cognitive effects of prucalopride on learning and memory tasks. In addition, the present findings suggest that prucalopride may reduce activation in the default mode network during task performance, which may be one mechanism by which prucalopride improves cognition. Future work should include a more detailed exploration of the breadth of cognitive changes produced by prucalopride and other 5-HT_4_ agonists, as well as of the duration of any effect considering the potential for desensitisation of 5-HT_4_ receptors. The effect of prucalopride on emotional processing in humans also requires further exploration with a higher dose to determine if the promising findings from animal studies may indeed translate into human investigations.

## Data Availability Statement

The datasets presented in this study can be found in online repositories. The names of the repository/repositories and accession number(s) can be found below: Code used for fMRI preprocessing and analysis is available at Gitlab (59) (https://git.fmrib.ox.ac.uk/acates/7dp_code). Anonymised behavioural data is available from OSF (10.17605/OSF.IO/JYH8K; https://osf.io/jyh8k/). Unthresholded fMRI group-level statistical maps will be made available on Neurovault. De-identified participant-level fMRI data will be made available on the WIN Open Data server. This is currently in development. Register here to find out when materials are available for download: https://web.maillist.ox.ac.uk/ox/subscribe/win-open-data. Further en-quires can be directed to the corresponding author AdeC, angharad.decates@psych.ox.ac.uk.

## Ethics Statement

The studies involving human participants were reviewed and approved by University of Oxford Central University Research Ethics Committee (MSD-IDREC reference R57219/RE001). The patients/participants provided their written informed consent to participate in this study.

## Author Contributions

SM, CH, AdeC, PC, and CG designed the study. LW, DG, and AdeC undertook data collection. AdeC, MM, CG, LC, LW, and DG conducted data analysis. AdeC, MM, LC, PC, CH, and SM interpreted the data. AdeC and MM drafted the manuscript. All authors contributed to revisions and approved the final draft.

## Author Disclaimer

The views expressed are those of the authors and not necessarily those of Wellcome, the NHS, the NIHR, or the Department of Health. None of these bodies had a significant role in the design, collection and analysis of data, or decision to publish this article.

## Conflict of Interest

CH has received consultancy fees from P1vital Ltd., Janssen Pharmaceuticals, Sage Therapeutics, Pfizer, Zogenix, Compass Pathways, and Lundbeck. SM has received consultancy fees from Zogenix, Sumitomo Dainippon Pharma, P1vital Ltd., and Janssen Pharmaceuticals. CH and SMhold grant income from Zogenix, UCB Pharma, and Janssen Pharmaceuticals. CH, SM, and PC hold grant income from a collaborative research project with Pfizer. The remaining authors declare that the research was conducted in the absence of any commercial or financial relationships that could be construed as a potential conflict of interest.

## Publisher’s Note

All claims expressed in this article are solely those of the authors and do not necessarily represent those of their affiliated organizations, or those of the publisher, the editors and the reviewers. Any product that may be evaluated in this article, or claim that may be made by its manufacturer, is not guaranteed or endorsed by the publisher.

## Footnotes

1
http://kdef.se/index.html

2
www.fmrib.ox.ac.uk/fsl
