# Supplementary material for: The Effect of the 5-HT4 Agonist, Prucalopride, on a Functional Magnetic Resonance Imaging Faces Task in the Healthy Human Brain
Source: Front Psychiatry. 2022 Apr 12;13:859123. doi: 10.3389/fpsyt.2022.859123 (PMC9039209; doi:10.3389/fpsyt.2022.859123)
Supplement: Supplementary file 1 [file Data_Sheet_1.docx]

##### **Supplementary Table 1: Sample demographics, baseline mood & personality**

| **Variable (measure)** | **Measurement** |  | **Placebo (N=21)** | **Prucalopride (N=22)** | **P value** | **Odds ratio (95% CI)** |
| --- | --- | --- | --- | --- | --- | --- |
| **Sex** | ***Number*** | ***Female*** | 13 | 13 | Ref | Ref |
|  |  | ***Male*** | 8 | 9 | 0.85 | 1.13 (0.33-3.83) ^[[1]](#footnote-1)^ |
| **Age** | ***Mean (SD)*** |  | 24.6 (3.9) | 24.7 (5.2) | 0.95 |  |
| **Education level** | ***Number*** | ***Postgraduate*** | 8 | 9 | Ref | Ref |
|  |  | ***Undergraduate*** | 4 | 4 | 0.89 | 0.89 (0.17-4.77) ^[[2]](#footnote-2)^ |
|  |  | ***6th form / equivalent*** | 9 | 9 | 0.86 | 0.89 (0.24-3.35) ^[[3]](#footnote-3)^ |
| **First language** | ***Number*** | ***English*** | 11 | 20 | Ref | Ref |
|  |  | ***Not English*** | 10 | 2 | 0.01*** | 0.11 (0.02-0.59) ^[[4]](#footnote-4)^ |
| **IQ (NART)** | ***Mean (SD)*** |  | 114.3 (6.9) | 115.9 (4.7) | 0.40 |  |
| **Handedness (EHI)** | ***Mean % right (SD)*** | | 84.7 (17.8) | 90.1 (15.4) | 0.30 |  |
| **Alcohol^a^** | ***Mean units/week (SD)*** | | 2.9 (2.6) | 4.0 (3.5) | 0.29 |  |
| **Caffeine^b^** | ***Mean drinks/day (SD)*** | | 1.7 (1.1) | 1.6 (1.3) | 0.77 |  |
| **Body mass index** | ***Mean (SD)*** |  | 23.1 (2.8) | 23.1 (2.4) | 0.95 |  |
| **Mood (BDI)** | ***Mean (SD)*** | | 0.9 (1.2) | 1.1 (1.2) | 0.70 |  |
| **Anhedonia(SHAPS)** | ***Mean (SD)*** | | 0.81 (1.7) | 0.59 (1.6) | 0.67 |  |
| **Anxiety (STAI-T)** | ***Mean (SD)*** | | 31.6 (7.5) | 29.1 (5.2) | 0.22 |  |
| **Personality (EPQ)** | ***Mean (SD) Neuroticism/Stability*** | | 4.6 (3.8) | 4.5 (3.2) | 0.91 |  |
|  | ***Psychotism/Socialisation*** | | 2.2 (1.9) | 2.1 (1.8) | 0.86 |  |
|  | ***Extroversion/Introversion*** | | 14.9 (3.7) | 13.8 (4.4) | 0.41 |  |
|  | ***Lie/Social desirability*** | | 10.6 (3.9) | 9.9 (4.4) | 0.58 |  |

(*** = <0.005; NART=National Adult Reading Test; EHI=Edinburgh Handedness Inventory; BDI=Beck Depression Inventory; SHAPS=Snaith-Hamilton Pleasure Scale; STAI-T=Spielberger Anxiety Inventory-Trait; EPQ=Eysenck Personality Questionnaire; ^a^=3 missing from placebo group; ^b^=1 missing from placebo group

**Supplementary Table 2: Questionnaire measures across visits**

|  | Placebo mean (SD)  (N=21) | Prucalopride mean (SD) (N=22) | P value (2dp) |
| --- | --- | --- | --- |
| Spielberger State Anxiety Inventory (STAI-S)  *Baseline*  *Research visit 1 (pre-Testing)* | 27.8 (8.1)  27.3 (6.0) | 24.8 (3.9)  28.6 (5.5) | 0.63 |
| Positive and Negative Affect Scale –  Positive (PANAS-P)  *Baseline*  *Research visit 1 (pre-Testing)* | 32.6 (5.1)  33.3 (5.6) | 34.4 (6.6)  32.6 (6.9) | 0.56 |
| Positive and Negative Affect Scale –  Negative (PANAS-N)  *Baseline*  *Research visit 1 (pre-Testing)* | 11.6 (2.3)  12.2 (3.1) | 10.6 (0.74)  11.2 (1.7) | Included in ANOVA above |

**Supplementary Figures:**

**Supplementary Figure 1. fMRI faces (emotional processing) task**


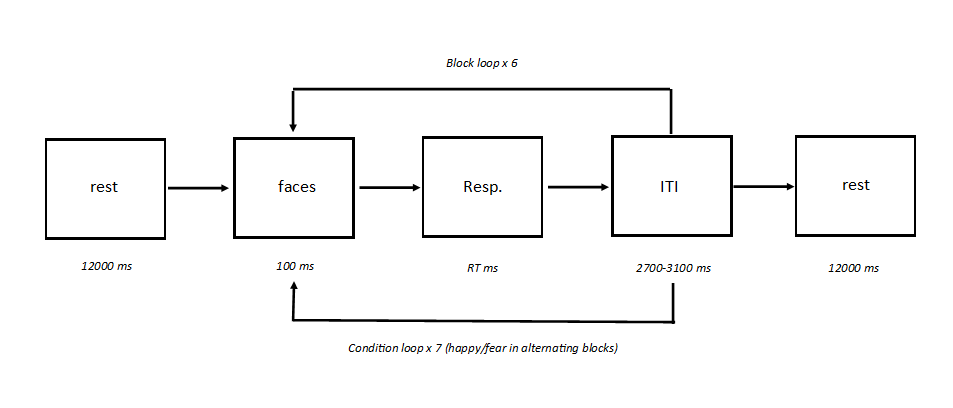


Resp. = response; ITI = inter-trial interval (routine duration controlled by the component code_jitter, which generates a random number between 2.7 and 3.1s and returns this as the variable)

**Supplementary Figure 2. Neural activity elicited by the fMRI Faces Task for all participants**

1. **Fearful Faces**

**
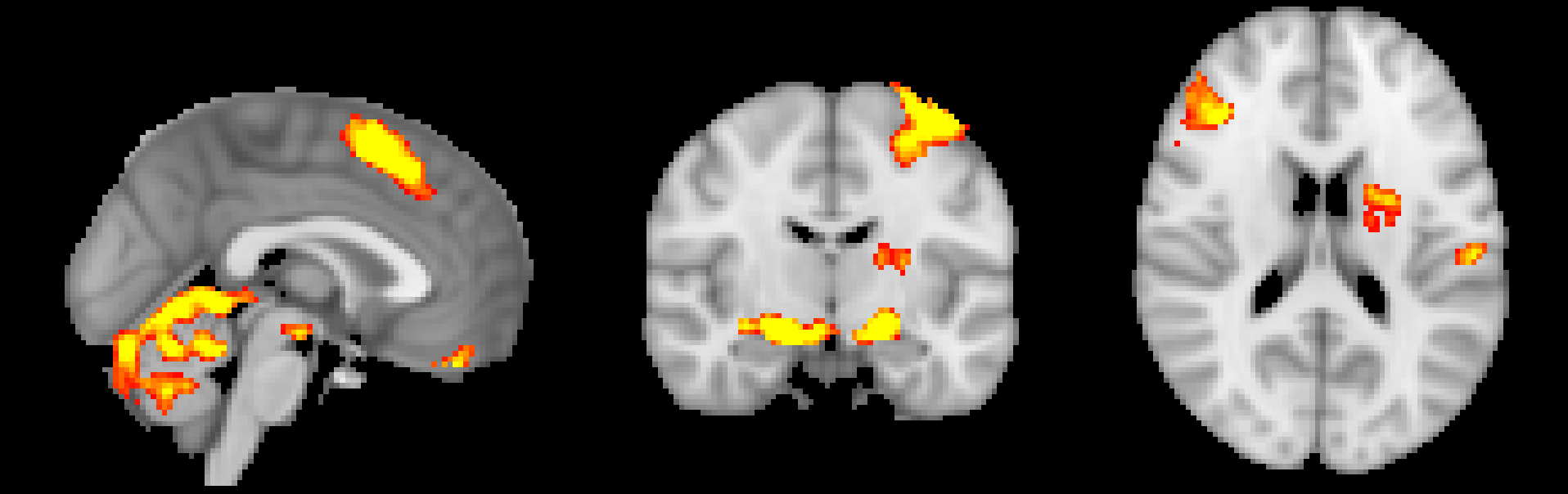
**

1. **Happy Faces**

**
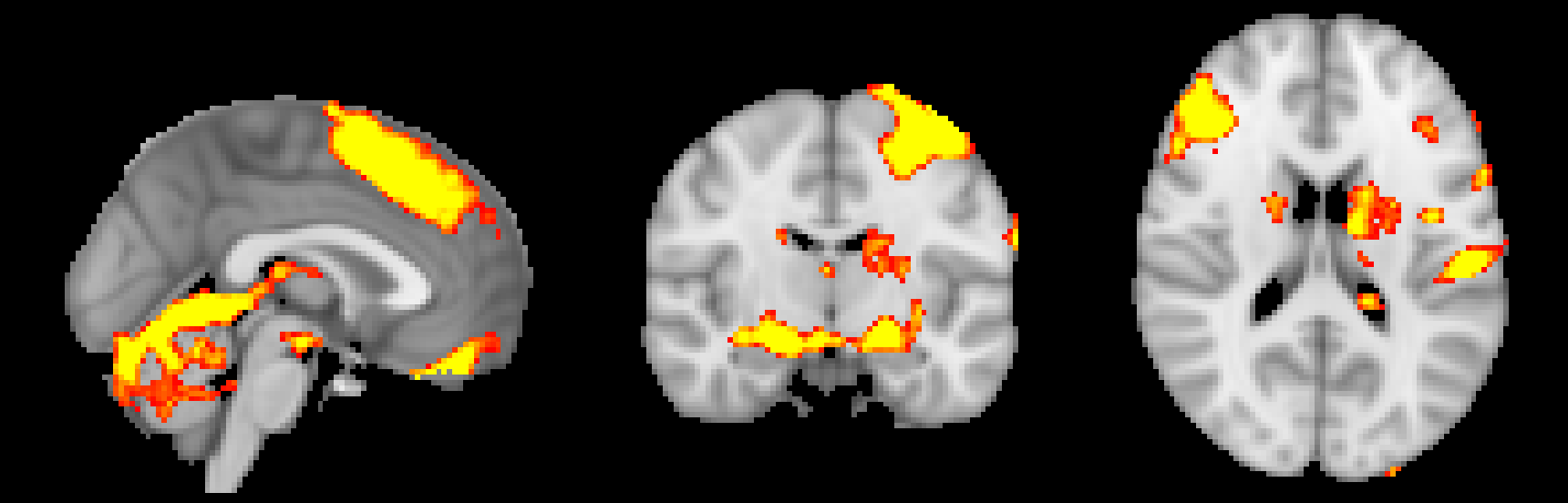
**

1. **Mean effect of task**

**
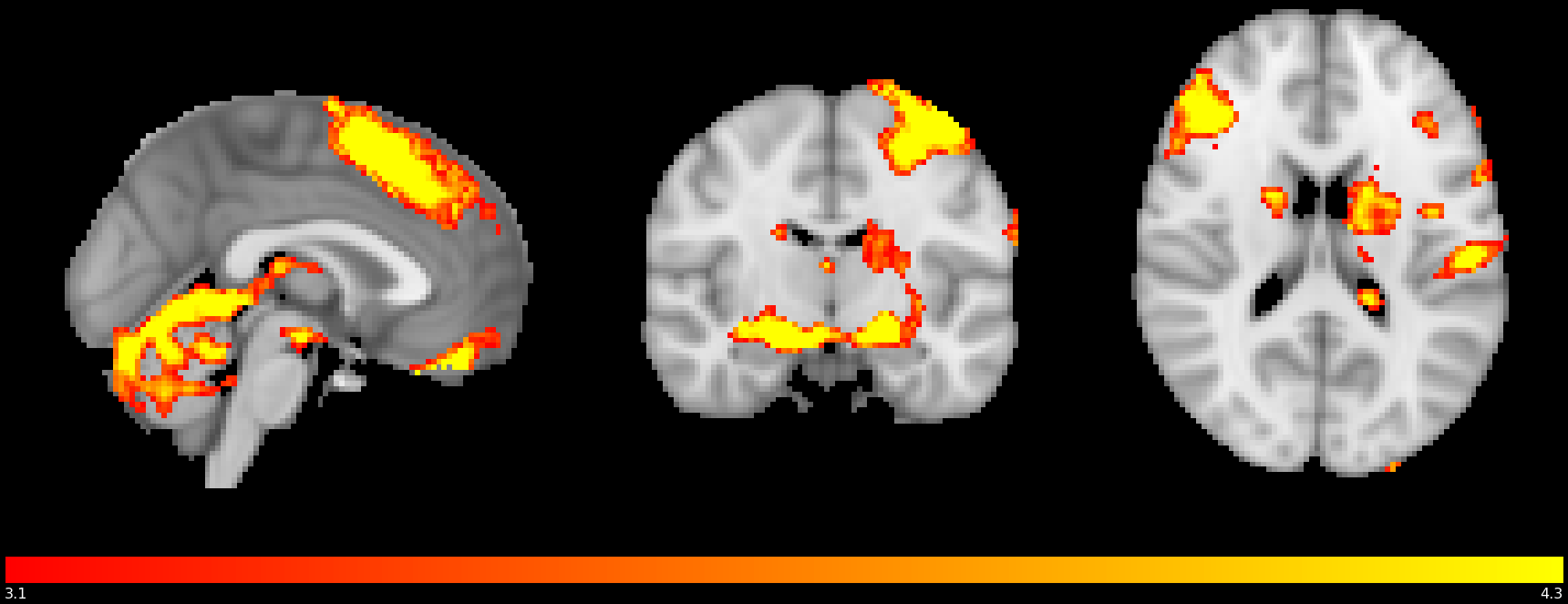
**

*Activation of brain areas during the fMRI faces task. The sagittal, coronal, and axial images identify neural activation (red) in response to (A) fearful faces versus fixation cross, (B) happy faces versus fixation cross, and (C) mean of both valences, across both treatment groups. Results are shown at the 3.1 cluster threshold significance level, corrected with FWE.*

**Supplementary Figure 3. Whole brain and ROI (ACC) results without correction for ASL and GM, and correction for gender only.**

A Corrected for gender B Uncorrected


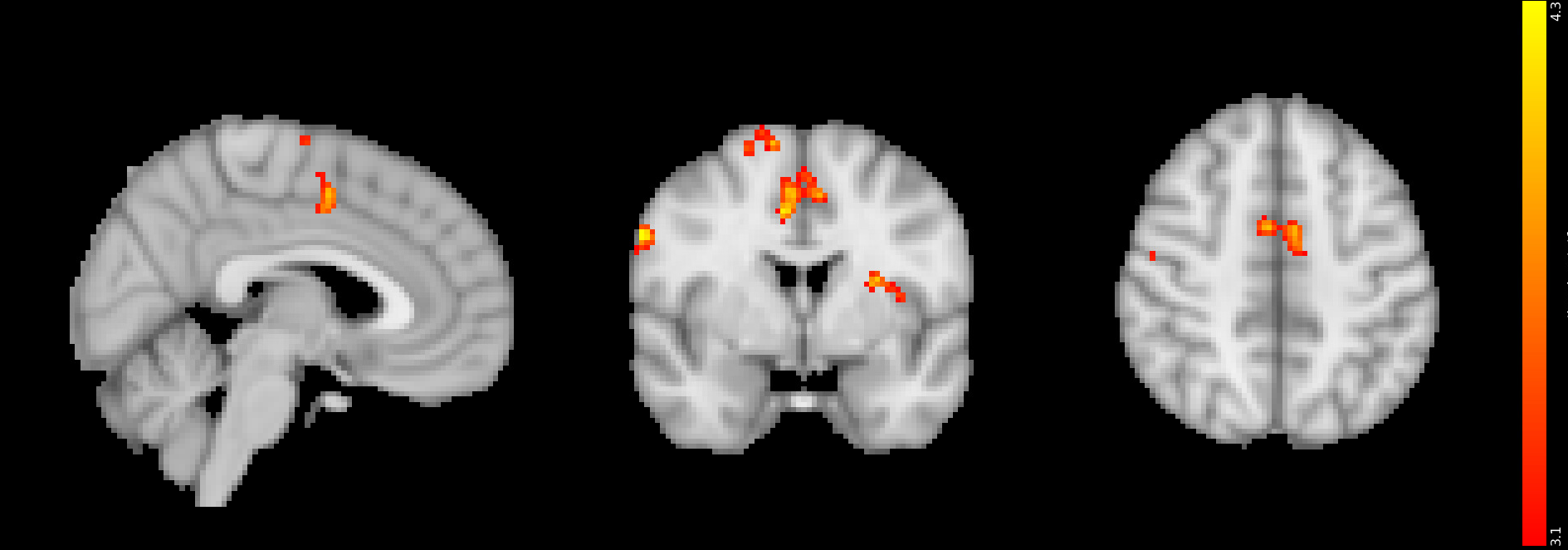

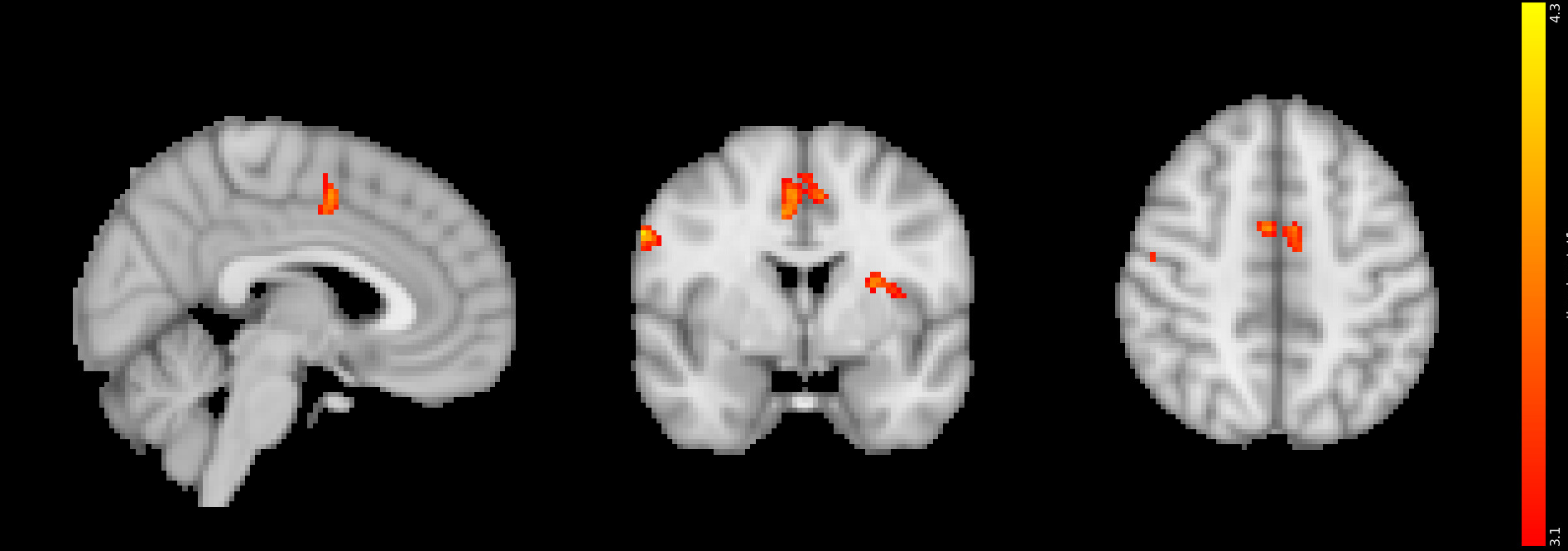


C D


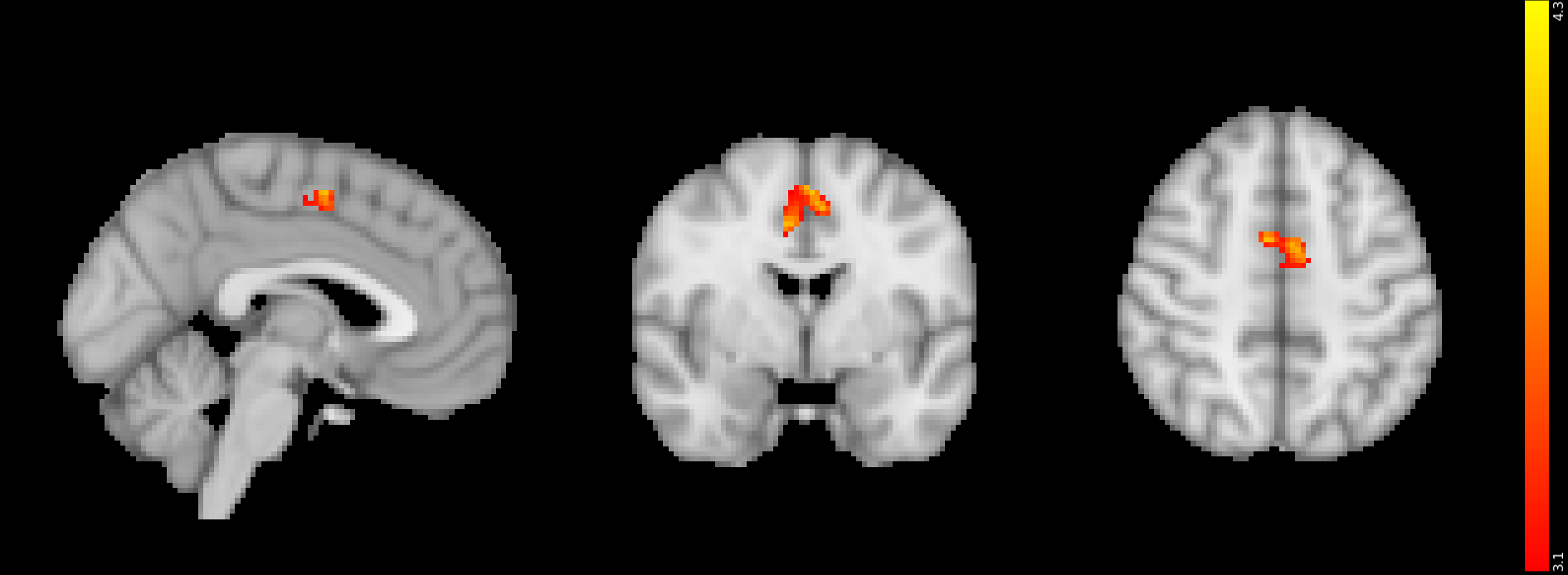

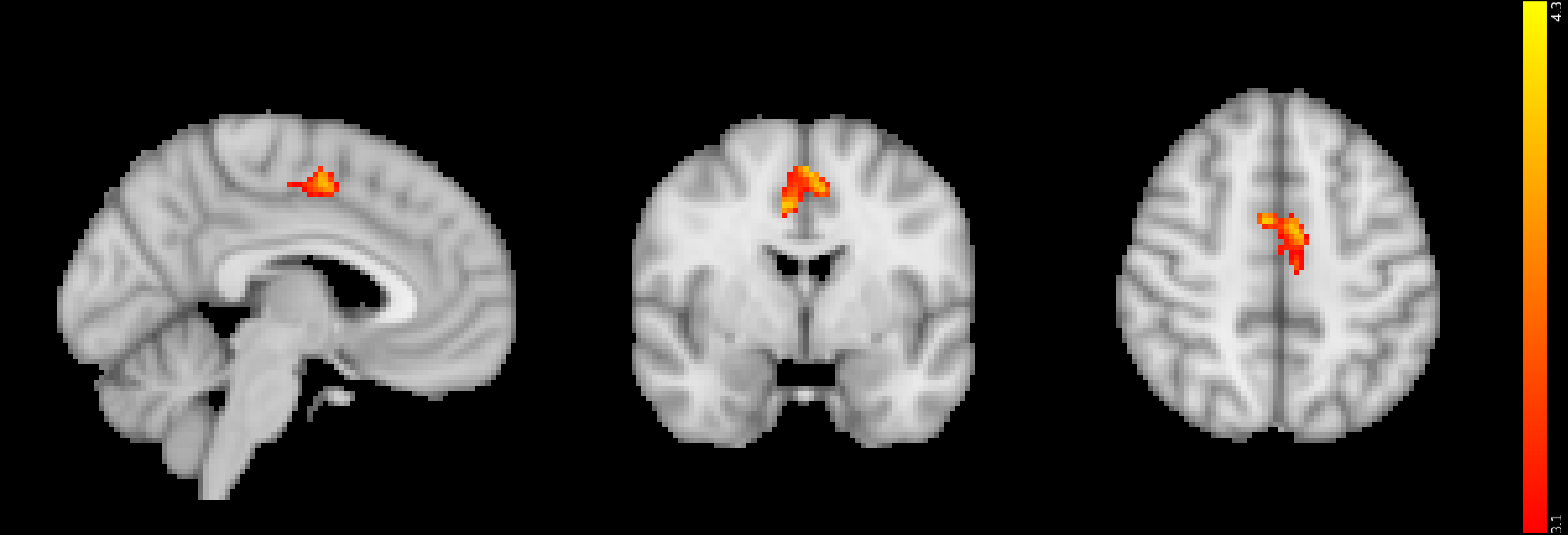


*A & B:*

*Whole-brain activation in response to mean effect of task (mean > baseline) in placebo vs. prucalopride group. Sagittal, coronal and axial images depicting significantly increased activation in the placebo group for the mean > fixation contrast in 6 clusters as per Figure 3 with A: corrected for gender only & B: uncorrected. Activation cluster patterns very similar to corrected for ASL + grey matter maps (as presented in Figure 1(A)). Images thresholded at z> 3.1, p<0.05 corrected. Red to yellow colours identify increases in brain activation (scale Z=3.1 to 4.3).*

*C & D:*

*Region of interest activation for the anterior cingulate cortex (ACC) in response to mean effect of task in placebo vs. prucalopride group. Sagittal, coronal and axial images depicting significantly increased activation in the placebo group for the mean > fixation contrast as per Figure 4. C: corrected for gender only; E: uncorrected. Signal change patterns very similar to corrected for ASL + grey matter maps (as presented in Figure 2(A)). Images thresholded at z> 3.1, p<0.05 corrected. Red to yellow colours identify increases in brain activation (scale Z=3.1 to 4.3). ACC Mask = Harvard Oxford atlas*

**Additional Supplementary Material**

Participant inclusion and exclusion criteria

| **Inclusion Criteria** |
| --- |
| Participant is willing and able to give informed consent for participation in the study  Not currently taking any medications (except the contraceptive pill)  Male or female  Aged 18-40 years  Sufficiently fluent English to understand and complete the task  Right handed  Body Mass Index in the range of 18-30 |
| **Exclusion criteria**  Not fluent in English  Any past or current Axis 1 DSM-V psychiatric disorder  Current usage of psychoactive medication (except the contraceptive pill, the Depo-Provera injection or the progesterone implant)  Current usage of any medication that will influence the MRI scan  Current or past history of drug or alcohol dependency  Currently pregnant or breastfeeding |

Study visits due to take place during the pre-menstrual week (female participants will be asked details of their menstrual cycle to schedule the study outside this week)

Not right handed

Body Mass Index outside the range of 18-30

History of cardiac, thyroid, or liver problems

An autoimmune disorder

Current, or a history of, gastro-intestinal disorder or irritable bowel syndrome

Epilepsy

Known lactate deficiency or any other problem absorbing lactose, galactose, or glucose

Participation in a study which uses the same computer tasks as those used in the present study

Participation in a study that involves the use of a medication within the last three months

Smoker > 5 cigarettes per day

Typically drinks > 6 caffeinated drinks per day

Any contraindication to MRI scanning (e.g. metal objects in your body, pacemakers, significant claustrophobia)

Full details of fMRI data acquisition and analysis

In this newer task version, participants generally found the gender discrimination behavioural part of the task more challenging than the previous version, with reduced accuracy compared to previous versions e.g. (Murphy *et al.*, 2009).

**MRI data acquisition**

fMRI acquisition: Encoding memory was assessed from a single run of 60 T2-weighted echoplanar imaging (EPI) slices covering the whole brain [repetition time (TR) 1200 ms, echo time (TE) 30 ms, flip angle 65°, field of view 216 mm, slice thickness 2 mm, voxel dimension 2mm isotropic, acquisition time 6min 28s]. Images were distortion corrected by an acquired fieldmap (echos at 4.92 and 7.38 ms, TR=590ms, flip angle = 46°).

Structural MRI acquisition: Additional high-resolution T1-weighted structural scans were acquired using a gradient echo sequence (TR 1900ms, TE 3.97ms, flip angle 8°, field of view 192mm, voxel dimension 1 mm isotropic, acquisition time 5min 31s) to allow later registration of the fMRI data into standard space.

ASL acquisition: Each participant also had a resting state pCASL perfusion-weighted scan with a 2D gradient spin echo readout and a PICORE Q2T labelling scheme. ASL data were collected as tag-control pairs with a TI of 1.8 seconds and a bolus duration of 0.7 seconds. ASL imaging parameters were: repetition time: 4100ms; minimum echo time: 14.0ms; FOV read: 220mm; FOV phase: 100%; voxel size: 3.4x3.4x4.5mm; 24 slices with 4.5mm thickness; echo spacing: 0.56mm; EPI factor: 64; post-labelling delays at: 250ms, 500ms, 750ms, 1000ms, 1250ms, and 1500ms; number of dynamics/repeats: 97 (1 volume was control); acquisition time = 6min 39s; fat saturation = on. A calibration image was acquired without labelling (TR = 6000ms).

Labelling plane was set with a time of flight neck scan (TR = 21ms, TE = 3.43ms, flip angle = 30°, field of view = 200mm, voxel dimension = 0.3 x 0.3 x 1.3 mm, acquisition time = 42s). Images were distortion corrected by an acquired fieldmap (echos at 4.92 and 7.38ms, TR=482ms, flip angle = 46°).

**MRI analysis**

fMRI task analysis: Imaging data were analysed with FSL ([www.fmrib.ox.ac.uk/fsl](http://www.fmrib.ox.ac.uk/fsl)).

fMRI data were pre-processed and analysed using FEAT (FMRI Expert Analysis Tool), version 6.0.4, part of FSL (FMRIB’s Software Library; www.fmrib.ox.ac.uk/fsl). DICOM (Digital Imaging and Communications in Medicine) files were downloaded from the server, checked for completeness, excessive movement and visual anomalies, and converted to a BIDS (Brain Imaging Data Structure)-standardised format nifti files using heudiconv (Halchenko, 2018) (heudiconv 0.5.4 (<https://github.com/nipy/heudiconv)>) before pre-processing. The structural anatomical scans were brain extracted using the Brain Extraction Tool (BET) (Smith, 2002). Formal MRI quality assessment was undertaken using the MRIQC package (Esteban *et al.*, 2017) (<https://mriqc.readthedocs.io/en/stable/index.html>), with data considered for rejection if it fell outside the normal range of values in the derived image quality metrics.

Pre-processing involved: motion correction using FMRIB’s Linear Image Registration Tool (FLIRT) (Jenkinson *et al.*, 2002); deletion of non-brain tissue using BET (Smith, 2002); spatial smoothing with a Gaussian kernel of 5 mm full-width-half-maximum; grand-mean intensity normalisation of the entire 4D dataset by a single multiplicative factor; high pass temporal filtering (Gaussian-weighted least-squares straight line fitting, with sigma of 90s) and B0 unwarping using fieldmap phase and magnitude images for distortion correction. No slice timing correction was applied. In addition, registration to high-resolution image and to a standard template [Montreal Neurological Institute (MNI)] was implemented using FNIRT nonlinear registration (Andersson, Jenkinson and Smith, 2007).

In the first-level analysis, individual activation maps were computed using the general linear model with local autocorrelation correction. Two explanatory variables were modelled: “happy” and “fear” images. Temporal derivatives were included in the model. Variables were modelled by convolving each block with a haemodynamic response function, using a variant of a gamma function (standard deviation 3s, mean lag 6s). No included participant demonstrated significant movement. At the whole-brain level, fearful images were contrasted with happy and fixation cross (baseline), resulting in the following model: 1) fear > fixation; 2) fear < fixation; 3) happy > fixation; 4) happy < fixation; 5) fear > happy; 6) happy < fear; 7) mean > fixation; 8) mean < fixation.

In the second-level analysis, whole-brain individual data were combined at a group level (participants on placebo vs. prucalopride) using a mixed-effects analysis, and cerebral blood flow and grey matter maps as covariates of no interest. Groups were contrasted with each other, resulting in the following comparisons: 1) placebo > prucalopride; 2) prucalopride > placebo; 3) placebo mean; 4) prucalopride mean; 5) mean of all participants. Brain activations showing significant group differences were identified at the whole-brain level using cluster-based thresholding (Z>3.1, family-wise error (FWE) p<0.05 corrected). Significant interactions from whole-brain analyses were further explored by extracting percentage BOLD signal change for each type of contrast. As the anterior cingulate cortex, medial frontal cortex, orbitofrontal cortex, and left or right amygdala and hippocampus were a particular focus, they were pre-specified as regions of interest (ROI). A functional ROI mask was created for each region by multiplying mean activation for each contrast of interest (on whole-brain data already corrected for multiple comparisons (FWE) and Z>3.1 as described above) for all participants by the Harvard-Oxford atlas anatomical mask at a 50% threshold. Percentage BOLD signal change for each contrast (in each hemisphere were relevant) was extracted in order to identify the profile of drug effect. All activations are reported using MNI co-ordinates.

FSLVBM, a voxel-based morphometry style analysis (Douaud *et al.*, 2007), was carried out to investigate potential grey matter differences between the two study-groups, underlying and potentially influencing group-related BOLD differences. Brain-extracted images (automatically created for each individual using FSLanat) were tissue-type segmented. Grey matter partial volume images were aligned to standard space using FLIRT and FNIRT registration tools. The resulting images were averaged, modulated and smoothed with an isotropic Gaussian kernel of 2mm to create a study-specific-template. A voxel-wise GLM was then applied using permutation non-parametric testing (5000 permutations). FSLFIRST (Patenaude *et al.*, 2011) was used to segment the left and right hippocampus for each participant, and the volume of individual hippocampi were determined using vertex analysis. This was then normalised for each individual’s brain volume and the resulting values for each hippocampus compared across groups using *t*-tests.

Regional and global blood flow was calculated for each individual. Distortion and motion corrected resting perfusion maps in units of mL/100g/min were calculated using Oxford_ASL (part of the Bayesian Inference for Arterial Spin Labelling (BASIL) tool, https://fsl.fmrib.ox.ac.uk/fsl/fslwiki/BASIL; (Chappell et al., 2009; Chappell et al., 2011) for each participant, which performs label-control subtraction, inference of voxelwise perfusion, and voxelwise calibration to obtain absolute perfusion maps, and controls for partial volume effects at the single subject level. FSL’s Anatomical Processing Script (FSL_Anat, https://fsl.fmrib.ox.ac.uk/fsl/fslwiki/fsl_anat) was used to pre-process each participant’s high resolution T1 structural image (includes bias-field correction, brain extraction and registration to standard space via FMRIB’s Linear Image Registration Tool (FLIRT) and FMRIB’s Non-linear Image Registration Tool (FNIRT). The processed perfusion images were non-linearly aligned with standard space via an initial linear transformation T1 structural space (using FLIRT), followed by application of the non-linear warp from fsl_anat. A Gaussian smoothing kernel of 2.12mm was applied to all the normalised images (to match functional data). Data were interrogated using voxel-wise generalized linear model (GLM) permutation nonparametric testing (5,000 permutations) with randomise (FSL’s tool for nonparametric permutation inference on neuroimaging data), correcting for multiple comparisons across space (cluster-based thresholding using TFCE and a family-wise error (FWE)-corrected cluster significance threshold of p<0.05 applied to the suprathreshold clusters). This results in spatial maps characterising the between-subject/group differences.

As post-hoc analyses, whole brain group feat results were also run using cerebral blood flow and grey matter maps as covariates of no interest, and sex as a covariate of interest. Anterior cingulate cortex perfusion between groups was compared using fslmeants: parameter estimates of perfusion were extracted from resting perfusion maps (previously computed using Oxford_ASL in units of ml/100g/min) using anatomical Harvard-Oxford masks at a 50% threshold.

References for supplementary material

Andersson, J., Jenkinson, M. and Smith, S. (2007) *Non-linear registration, aka Spatial normalisation.*: FMRIB technical report TR07JA2.

Chappell, M. A., Groves, A. R., MacIntosh, B. J., Donahue, M. J., Jezzard, P. and Woolrich, M. W. (2011) 'Partial volume correction of multiple inversion time arterial spin labeling MRI data', *Magn Reson Med,* 65(4), pp. 1173-83.

Chappell, M. A., Groves, A. R., Whitcher, B. and Woolrich, M. W. (2009) 'Variational Bayesian inference for a nonlinear forward model', *Trans. Sig. Proc.,* 57(1), pp. 223-236.

Douaud, G., Smith, S., Jenkinson, M., Behrens, T., Johansen-Berg, H., Vickers, J., James, S., Voets, N., Watkins, K., Matthews, P. M. and James, A. (2007) 'Anatomically related grey and white matter abnormalities in adolescent-onset schizophrenia', *Brain,* 130(Pt 9), pp. 2375-86.

Esteban, O., Birman, D., Schaer, M., Koyejo, O. O., Poldrack, R. A. and Gorgolewski, K. J. (2017) 'MRIQC: Advancing the automatic prediction of image quality in MRI from unseen sites', *PLoS One,* 12(9), pp. e0184661.

Author (2018) *nipy/heudiconv v0.5.4* (Version <https://doi.org/10.5281/zenodo.3760062>).

Jenkinson, M., Bannister, P., Brady, M. and Smith, S. (2002) 'Improved optimization for the robust and accurate linear registration and motion correction of brain images', *Neuroimage,* 17(2), pp. 825-41.

Murphy, S. E., Norbury, R., O'Sullivan, U., Cowen, P. J. and Harmer, C. J. (2009) 'Effect of a single dose of citalopram on amygdala response to emotional faces', *Br J Psychiatry,* 194(6), pp. 535-40.

Patenaude, B., Smith, S. M., Kennedy, D. N. and Jenkinson, M. (2011) 'A Bayesian model of shape and appearance for subcortical brain segmentation', *Neuroimage,* 56(3), pp. 907-22.

Smith, S. M. (2002) 'Fast robust automated brain extraction', *Hum Brain Mapp,* 17(3), pp. 143-55.

1. OR of being male in prucalopride group, compared to females [↑](#footnote-ref-1)
2. OR of being undergraduate in prucalopride group, compared to postgraduate [↑](#footnote-ref-2)
3. OR of being 6th form in prucalopride group, compared to postgraduate [↑](#footnote-ref-3)
4. OR of being non-English in prucalopride group, compared to English [↑](#footnote-ref-4)
